# Not Substitutes for Mothers

**Published:** 1920-06-12

**Authors:** 


**THE PUBLIC WELL-BEING—(*continued*).**

### Not Substitutes for Mothers.

A lively discussion on the care of babies in nursery schools took place at the conference of the National Association of Head Teachers at York. There was general approval of the inclusion of nursery schools in the scheme of educational reconstruction, but a Birmingham delegate threw a bombshell into the midst of this by declaring that nursery schools were absurd in conception and immoral in their consequences. He was immediately pounced upon from all sides, especially by the lady teachers, whose approval of the schools was attributed by him largely to a disappointed maternal instinct. His argument was better than his rather violent expression of it. The freer the child is up to seven years of age the better; the proper place for the mothers is the home, yet the schools would enable them to feel at liberty to go to work in factories, thus endangering the proper atmosphere of the home. As propositions these

THE PUBLIC WELL-BEING—(*continued*).

points are not negligible, but they are not altogether relevant. If, as the conference was disposed to suggest, the schools are looked upon more as nurseries than as schools (extending to those who may not be able to afford them the privileges of nurseries in a collective form), then there is no reason to suppose the children will get less freedom there than in their own homes. The fact has also to be faced that, though the mother's place is certainly in the home, and not in the factory, many mothers with large families and no assistance are greatly overworked. To them the schools are an immense relief, and for the few hours daily the children probably have a better time.

A Leeds teacher (Miss Harrison) added to some good points in favour of the schools the statement that if it came to a choice between the mother who "merely loved her child in an animal way" and the sympathetic teacher—single woman, perhaps—who loved babies, then the child was certainly better off in the care of the teacher. We are not so sure, however, that "animal motherhood" ought thus lightly to be set aside. There is immense power in it, and not even the sympathy of a single-woman teacher can take its place. People who love other mothers' babies can be very eclectic in their tastes, and it would take a very sympathetic teacher to embrace the lot with a maternal instinct equivalent to, perhaps, a score of mothers. We have no use for nursery schools conceived in an endeavour to improve on good motherhood, but schools to assist good motherhood where it is needed, and to help save the tiny children from some of the consequences of neglectful motherhood can be of great physical, mental, and social value within those lines.

